# Eculizumab for Thrombotic Microangiopathy Associated with Antibody-Mediated Rejection after ABO-Incompatible Kidney Transplantation

**DOI:** 10.1155/2017/3197042

**Published:** 2017-12-28

**Authors:** Luca Lanfranco, Melanie Joly, Arnaud Del Bello, Laure Esposito, Noelle Cognard, Peggy Perrin, Bruno Moulin, Nassim Kamar, Sophie Caillard

**Affiliations:** ^1^Department of Nephrology and Organ Transplantation, CHU Rangueil, Toulouse, France; ^2^Department of Nephrology and Transplantation, University Hospital of Strasbourg, Strasbourg, France; ^3^Université Paul Sabatier, Toulouse, France; ^4^INSERM U1043, IFR-BMT, CHU Purpan, Toulouse, France

## Abstract

Thrombotic microangiopathy is a form of antibody-mediated rejection (ABMR): it is the main complication of ABO-incompatible (ABOi) kidney transplantation (KT). Herein, we report on two cases of ABMR with biological and histological features of thrombotic microangiopathy (TMA) that were treated by eculizumab after ABOi KT. The first patient presented with features of TMA at postoperative day (POD) 13. Because of worsening biological parameters and no recovery of kidney function, despite seven sessions of immunoadsorption, a salvage therapy of eculizumab was started on POD 23. Kidney function slightly improved during the first 4 months after transplantation. Eculizumab was stopped at month 4. However, kidney function worsened progressively, leading to dialysis at month 13 after transplantation. The second patient presented with features of TMA at POD 1. In addition to immunoadsorption therapy, eculizumab was started on POD 6. Kidney function improved. Eculizumab was stopped on POD 64 and immunoadsorption sessions were stopped on POD 102. At the last follow-up (after 9 months), eGFR was at 43 mL/min/1.73 m^2^. Our case reports show the beneficial effect of eculizumab to treat ABMR after ABOi KT. However, it should be given early after diagnosing TMA associated with ABMR.

## 1. Introduction

Mid- and long-term kidney allograft survival rates have been shown to be similar after living-donor ABO-compatible (ABOc) and ABO-incompatible (ABOi) kidney transplantation (KT) [1–3]. In order to ensure successful ABOi KT, the titers of isoagglutinins that are directed against the donor should be decreased: apheresis and rituximab are used to decrease isoagglutinin levels.

Antibody-mediated rejection (ABMR) remains the main complication of ABOi KT. Thrombotic microangiopathy (TMA) is a form of ABMR. It is still a matter of debate whether ABMR is mediated by natural (IgM) or immune (IgG) isoagglutinins. In both cases, ABMR occurs through the activation of the complement system [4]. Eculizumab, a monoclonal anti-C5 antibody that blocks the complement cascade, was used to prevent or treat refractory ABMR after ABOc [5–7] or ABOi KT [8, 9]. Herein, we report on two cases of ABMR with biological and histological features of TMA that were treated with eculizumab after ABOi KT.

## 2. Case 1

A 64-year-old man (group O) received a first ABOi living unrelated-donor KT for nephroangiosclerosis. The donor was his spouse (group AB). Before, at, and after transplantation, anti-HLA antibodies were assessed by the Luminex SA assay and remained negative. At baseline, anti-A and anti-B isoagglutinin titers that were assessed using hemagglutination were 1/80 and 1/10, respectively. The desensitization protocol included eight nonspecific immunoadsorption sessions (Adasorb® reusable columns, Fresenius, Bad Homburg, Germany), two specific-IA sessions (Glycorex® column, Lund, Sweden), and rituximab (375 mg/m^2^ given for 30 days before transplantation). At transplantation, both anti-A and anti-B isoagglutinin levels were at 1/5. Basiliximab induction therapy was given on days 0 and 4 (20 mg each). Tacrolimus, mycophenolic acid, and prednisolone were started 12 days before transplantation and were continued thereafter.

**Figure 1:**
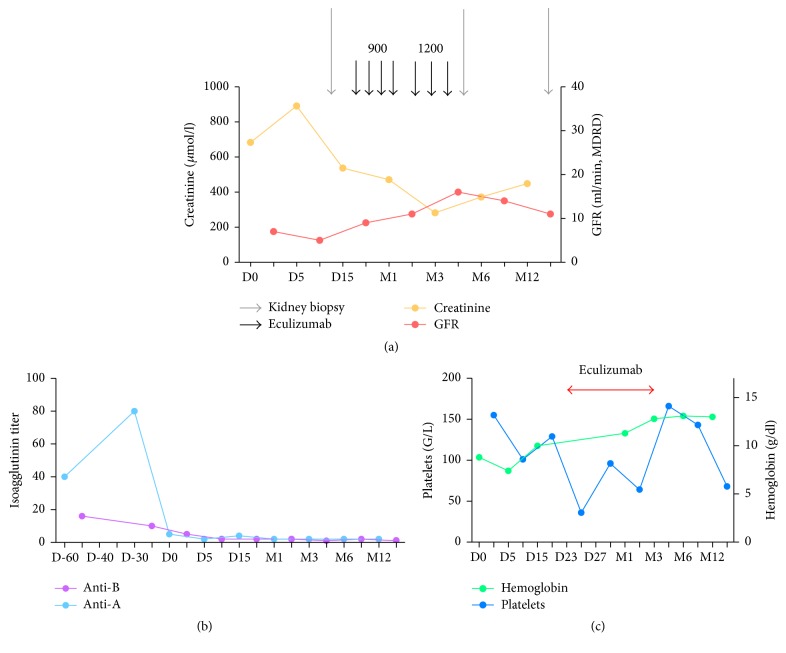
Kidney function (a), isoagglutinin titers (b), and hematological parameters (c) in patient 1. Evolution after kidney transplantation.

At postoperative day (POD) 3, the patient presented with partial kidney allograft venous thrombosis that required surgery, and the graft was reimplanted. Consequently, the patient recovered diuresis, but no graft function. On POD 13, a kidney biopsy was performed and showed the presence of thrombotic microangiopathy without microcirculation inflammation and no features of T-cell mediated rejection. C4d staining was positive. At that time, serum creatinine level was 546 *μ*mol/L (Figure 1). Hemoglobin level was 10.1 g/dL. Schistocyte count was <15‰. Lactate dehydrogenase was 636 IU/L. Platelet count had decreased from 155,000/mm^3^ at transplantation to 116,000/mm^3^. Tacrolimus trough level was 9.7 ng/mL. Anti-A and anti-B isoagglutinin levels stayed at 1/5. Complement fraction levels were within the normal ranges. No autoimmune disease or phospholipid syndrome was evidenced. Classical viral infections were ruled out. Genetic analysis for the complement pathway ruled out atypical HUS.

We concluded treatment with isoagglutinin-induced ABMR. Hence, we started apheresis using plasma exchanges (*n* = 3) followed by nonspecific IA sessions (*n* = 4). Because of a worsening of platelet count (36,000/mm^3^ at day 22) and no recovery of kidney function, a salvage therapy using eculizumab was decided upon. It was started on day 23 at a dose of 900 mg per week for 4 weeks and then 1200 mg every 2 weeks. The platelet count rapidly increased to 96,000/mm^3^ (day 29). Hemoglobin level slightly improved and the patient recovered modest kidney function, which permitted dialysis to be stopped at POD 30 (SCr of 460 *μ*mol/L, eGFR CKD Epi 11 mL/min). Kidney function improved during the first 4 months after transplantation and platelet counts returned within the normal range.

At month 4, a control kidney biopsy was performed: it revealed no features of cellular or ABMR, but sequelae of the previous TMA episode (t0, i0, g0, v0, ptc0, cg0, cv0, ci2, and ct2). Hence, eculizumab was stopped at month 4. Between months 4 and 12, platelet count remained within the normal range and isoagglutinin levels were unchanged. Conversely, kidney function remained impaired and worsened progressively. At month 13 after transplantation, he started dialysis again.

## 3. Case 2

A 48-year-old man (group O) received a second ABOi living unrelated-donor KT for IgA nephropathy. The donor was his spouse (group A). No anti-HLA donor specific antibodies (DSA) at or after transplantation were detected. At baseline, natural and immune anti-A isoagglutinins titers were 1/64 each (using hemagglutination). The desensitization protocol included six immunoadsorption sessions (Immunosorba, Fresenius, Bad Homburg, Germany) and rituximab (375 mg/m^2^ given 34 days before transplantation). At transplantation, natural and immune anti-A titers were 1/8 and 1/16, respectively. An antithymocyte globulin induction therapy was given from days 0 to 4 (1.3 mg/kg/d). Tacrolimus, mycophenolic acid, and prednisolone were started at 14 days before transplantation and were continued thereafter.

**Figure 2:**
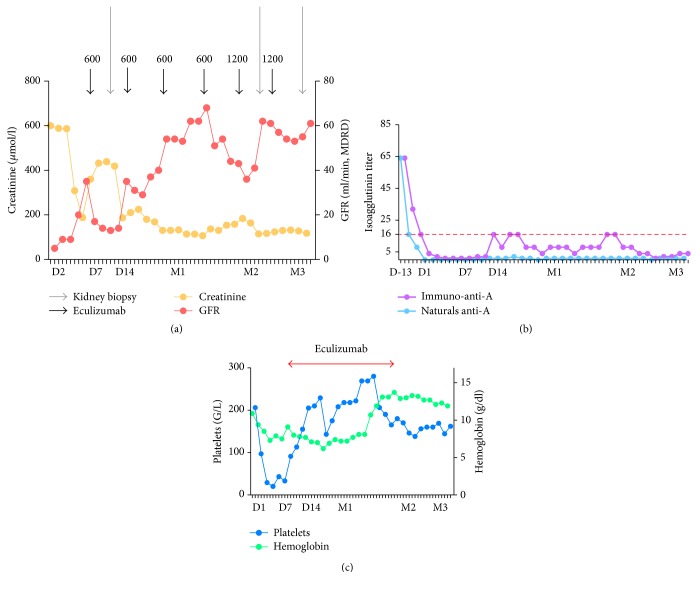
Kidney function (a), isoagglutinin titers (b), and hematological parameters (c) in patient 2. Evolution after transplantation.

After transplantation, the patient recovered diuresis without a decrease in serum creatinine levels. At POD 1, the patient presented with biological features of TMA, including hemolytic anemia, thrombocytopenia (platelet count at 49,000/mm^3^), and no improvement in kidney function (Figure 2). Anti-A level was 1/4. Anti-A-mediated ABMR was suspected and plasmapheresis was initiated: a daily session was given between PODs 1 and 4 and then 3 times weekly until POD 42.

**Figure 3:**
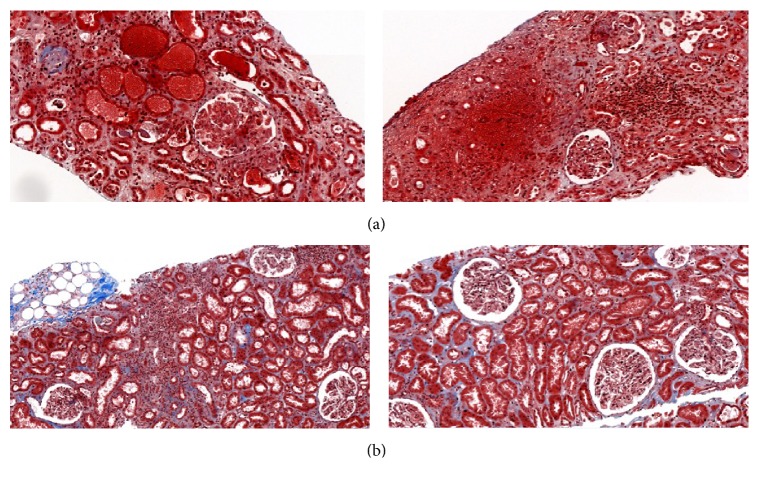
(a) Kidney allograft biopsy at postoperative day 12 in patient 2: thrombotic microangiopathy lesions, antibody-mediated rejection (t0, i0, g3, and cpt2). (b) Kidney allograft biopsy at postoperative day 54 in patient 2. Lesions of mixed acute cellular and antibody-mediates rejection (i2 i2 ti2 g2 cpt2), IF/TA 1, and no more lesions of thrombotic microangiopathy.

Due to the severity of the clinical and biological presentation, eculizumab was given at a dose of 900 mg on POD 6, 17, 24, and 36 and then of 1200 mg on POD 50 and 64. A first kidney biopsy was done on POD 12 (Figure 3(a)) and revealed thrombotic microangiopathy, glomerulitis (g3), peritubular capillaritis (cpt2), and a peritubular C4d deposition. No histologic sign of acute cellular rejection was seen. Rapidly after initiation of eculizumab, hematological parameters improved, as well as kidney function (Figure 2). Hemodialysis sessions were stopped at POD 16. The patient was discharged at POD 37. At that time, serum creatinine level was at 114 *μ*mol/L (eGFR 62 mL/min/1.73 m^2^). During the first month after transplantation, natural isoagglutinin anti-A remained below 1/4 and immune isoagglutinin anti-A increased transiently to 1/16. ADAMTS 13 activity was normal (81%), and genetic analysis for the complement pathway ruled out atypical HUS.

At POD 53, serum creatinine levels increased from 110 to 154 *μ*mol/L, with increasing immuno-anti-A antibody titers, from 1/8 to 1/16, but without biological features of TMA. A second graft biopsy showed an acute mixed rejection (t2 i2 ti2 g2 cpt2) without TMA lesions (Figure 3(b)). Three methylprednisolone pulses were given and immunoabsorption sessions were restarted (2 session/week for 6 weeks). Eculizumab was stopped at POD 64 and immunoadsorption sessions were stopped at POD 102. Kidney function improved. A third graft biopsy was performed on POD 102. It did not reveal any sign of rejection or TMA. At the last follow-up (after 9 months), serum creatinine level was 160 *μ*mol/L (eGFR 43 mL/min/1.73 m^2^).

## 4. Discussion

ABMR is a well-known complication of ABOi kidney transplantation. Most AMR episodes are associated with activation of the complement pathway. ABMR is mediated by the classical pathway cleavage of C3 into C3b and C3a and the downstream formation of the membrane-attack complex, sC5b-9. Eculizumab is a humanized anti-C5 monoclonal antibody that blocks C5b-9 and C5a formation, which prevents the proinflammatory, prothrombotic, and lytic functions of complement [10]. Eculizumab is approved for treatment of paroxysmal nocturnal hemoglobinuria and atypical hemolytic and uremic syndrome (aHUS). Because of its mechanism of action to target the complement pathway, it has been also used in desensitization protocols to prevent ABMR [11, 12]. Although the incidence of ABMR was decreased in patients given eculizumab-based desensitization protocols, compared to those having not received eculizumab [11], at one year, chronic AMR lesions were similar in both groups [12].

Eculizumab has been also used to treat ABMR in ABO-compatible [5–7] and ABOi kidney-transplant patients [8, 9], as well as after ABOi pancreas [8] and heart transplantation [13]. In addition,* in vitro* and* in vivo*, eculizumab was found to inhibit hemolytic reaction after ABO-incompatible red blood-cell transfusion [14, 15].

Biglarnia et al. have reported a case of an intentional ABOi deceased-donor kidney and pancreas transplantation with severe ABMR that occurred at POD 9 during a rebound of isoagglutinins, despite immunoadsorption treatment. The use of two doses of eculizumab (600 mg at PODs 10 and 14) successfully treated the acute episode. At 6 months after transplantation, both kidney and pancreatic functions were normal [8].

Stewart et al. have reported a case of accelerated ABMR that occurred very early after ABOi KT, caused by a rebound in isoagglutinin levels [9]. Biological and histological features of TMA were observed. In the absence of any improvement, despite plasmapheresis, rituximab, intravenous immunoglobulins, and splenectomy, eculizumab was given as a salvage therapy at PODs 8, 10, 12, 14, and 21 [9]. The patient recovered kidney function and had a functional graft at 1 year after transplantation.

Recently, Ikeda et al. have reported two cases of TMA that occurred very early after ABOi kidney transplantation [16]. In both cases, patients had biological and histological features of TMA. Eculizumab was given for 2 and 3 months, respectively, in addition to intravenous immunoglobulins, rituximab, and a few sessions of plasmapheresis (2 and 4 sessions, resp.) [16]. In both cases, biological features of TMA disappeared and kidney function recovered [16].

Similar to the four previous published cases [8, 9, 16], our two patients presented with ABMR and biological features of TMA very early after transplantation. Both were initially treated by apheresis, and eculizumab was added thereafter. Similar to previous published cases [8, 9, 16], in one of the two cases (case 2), a rebound (slight and delayed) in isoagglutinin level was observed. It is important to note that, in the first case, immune isoagglutinin levels were not assessed at that time. In both cases, features of TMA regressed rapidly. The second patient had good kidney function at the last follow-up whereas the first patient had lost his kidney allograft at month 13 after transplantation. This can be explained by the fact that eculizumab was started at POD 23 in the first case, which was much later than for the second patient (POD 6) and the time-periods reported in previous reports [8, 9, 16].

In aHUS, it has been shown that early initiation of eculizumab was associated with greater improvement in kidney function [17]. Finally, it is important to note that eculizumab can fail to prevent [18] and treat ABMR [7], suggesting that there may be other pathways involved in the ABMR mechanisms.

To summarize, we describe the beneficial effect of eculizumab to treat ABMR after ABOi KT. We suggest that eculizumab should be given very early in ABOi KT patients presenting biological and histological features of TMA than can be related to ABMR.
